# Pneumonia Patients Caused by Co-infection With SARS-CoV-2 and Human Adenovirus in China

**DOI:** 10.3389/fmed.2021.735779

**Published:** 2021-09-28

**Authors:** Shaofu Qiu, Ge Zeng, Peihan Li, Xiaohui Li, Hongbo Liu, Xinying Du, Hongbo Liu, Heng Zhang, Xingyu Xiang, Hui Wang, Xiangbing Chen, Guangyao Yang, Sai Tian, Ligui Wang, Mingjuan Yang, Chaojie Yang, Lidong Gao, Shixiong Hu, Hongbin Song, Zhifei Zhan

**Affiliations:** ^1^Chinese PLA Center for Disease Control and Prevention, Beijing, China; ^2^Hunan Provincial Center for Disease Control and Prevention, Workstation for Emerging Infectious Disease Control and Prevention, Chinese Academy of Medical Sciences, Changsha, China; ^3^Wangcheng Center for Disease Control and Prevention, Changsha, China; ^4^Changsha Center for Disease Control and Prevention, Changsha, China

**Keywords:** SARS-CoV-2, HAdV, co-infection, metagenomic sequencing, presymptomatic transmission

## Abstract

**Objectives:** To data, no patients with obvious epidemiological relationship co-infected with SARS-CoV-2 and other pathogens have been reported. Here, we investigated 10 patients caused by co-infection with SARS-CoV-2 and human adenovirus (HAdV), resulting in third-generation transmission.

**Materials and Methods:** From Jan 15, 2020, we enrolled 10 patients with pneumonia in Hunan Province, China. Epidemiological, clinical, and laboratory investigation results from these patients were analyzed. An epidemiological investigation was performed to assess whether patient infections were linked using conventional methods and metagenomic sequencing.

**Results:** The presence of co-infection with SARS-CoV-2 and HAdV was determined via RT-PCR and metagenomic sequencing. Phylogenetic analysis revealed that SARS-CoV-2 and HAdV genomes clustered together, with similar genetic relationships. The first patient likely became co-infected during meetings or travel in Wuhan. The patient transmitted the virus via dinners and meetings, which resulted in four second-generation cases. Then, a second-generation case transmitted the virus to her family members or relatives via presymptomatic transmission.

**Conclusions:** This study described an example of co-infection with SARS-CoV-2 and HAdV in pneumonia patients, which caused third-generation cases and inter-regional transmission via meetings, household interactions, and dinner parties. We also observed the persistent and presymptomatic transmission of co-infection, which has the potential to make the continued control of the COVID-19 pandemic challenging. Continuous surveillance is needed to monitor the prevalence, infectivity, transmissibility, and pathogenicity of SARS-CoV-2 co-infection with other pathogens to evaluate its real risk.

## Introduction

The coronavirus disease 2019 (COVID-19) pandemic caused by severe acute respiratory syndrome coronavirus 2 (SARS-CoV-2) is ongoing (1, 2). As of July 2, 2021, data from the WHO have shown that a total of 182,319,261 confirmed cases of COVID-19 have been reported worldwide, including 3,954,324 deaths (https://covid19.who.int/). Thus, the virus continues to be a serious public health problem globally.

The COVID-19 outbreak was first reported in Wuhan, China on December 31, 2019, and was associated with the Huanan Seafood Wholesale Market (1, 2). It has been reported that 5 million people emigrated from Wuhan to other regions of China and other countries before the city was locked down, a situation that likely allowed for the spread of the virus (3, 4). SARS-CoV-2 infections among medical workers and family clusters have been identified, and human-to-human transmission of SARS-CoV-2 occurs largely in families in China (5–7). SARS-CoV-2 co-infection alongside other pathogens have been reported, including occasional co-infections involving human adenovirus (HAdV) and SARS-CoV-2. However, reports of co-infections are rare (8–12). Moreover, little is known regarding whether SARS-CoV-2 co-infections are transmitted between individuals at rates capable of causing clusters of acute respiratory infections. Here, using conventional and genomic epidemiological methods, we investigated a clustered pneumonia patients caused by co-infection with SARS-CoV-2 and HAdV, resulting in a large-scale transmission and even third-generation human-to-human transmission.

## Materials and Methods

### Patients and Samples

In January of 2020, we enrolled 10 patients with fever, respiratory symptoms, and pulmonary infiltrates. Among them, three patients were admitted to a local hospital in Huaihua City, Hunan Province, China, and seven were admitted to another hospital in Shaoyang City, Hunan Province. Due to the serious outbreak of SARS-CoV-2 in Wuhan, a joint team comprising staff from provincial and municipal Centers for Disease Control and Prevention (CDC) conducted detailed field investigations of all suspected SARS-CoV-2 cases. Data were collected via standardized forms through interviews with infected persons, relatives, and close contacts. We recorded and analyzed the epidemiological, clinical, laboratory, and microbiological investigation results from the patients. Throat swab samples were collected from patients and their close contacts, and were stored in viral transport media for further identification. This study was approved by the institutional review board of the participating units, and written informed consent was obtained from all patients.

### Laboratory Identification of Patient Samples

Respiratory samples from patients with suspected infections and their contacts were initially detected for the presence of SARS-CoV-2 at the local CDC facility, using real-time reverse-transcriptase PCR (RT-PCR) assays (Sansure Biotech, Changsha, Hunan, China). Samples positive for SARS-CoV-2 were sent to our laboratory for further identification. Nucleic acids were extracted using the TIANamp Virus DNA/RNA Kit (Tiangen, Beijing, China), and tested for the presence of SARS-CoV-2, HAdV, influenza A virus and influenza B virus via RT-PCR assays (Shanghai BioGerm Medical Biotechnology Co., Ltd., Shanghai, China).

### Whole-Genome Sequencing and Bioinformatics Analysis

Metagenomic sequencing was performed directly on throat swab samples using the Illumina Novaseq platform, and de novo assembly was performed using SPAdes v3.13.0 (13). Sanger sequencing was used to close gaps and verify ambiguous sequences. Representative Betacoronavirus genomes, including that of SARS-CoV-2, and genomes of HAdV species B, including HAdV serotype 3 (HAdV-3), were retrieved from GenBank, and multiple sequence alignments were performed using Mafft 7.450 (14). Phylogenetic analysis was performed using Mega 7.0.21 (15), with the maximum likelihood method with 1,000 bootstrap replicates. Single nucleotide variants were analyzed using Bwa 0.7.17-r1188 (16) and Samtools 1.9 (17).

## Results

### Epidemiological Investigation of the Patients

**Figure 1:**
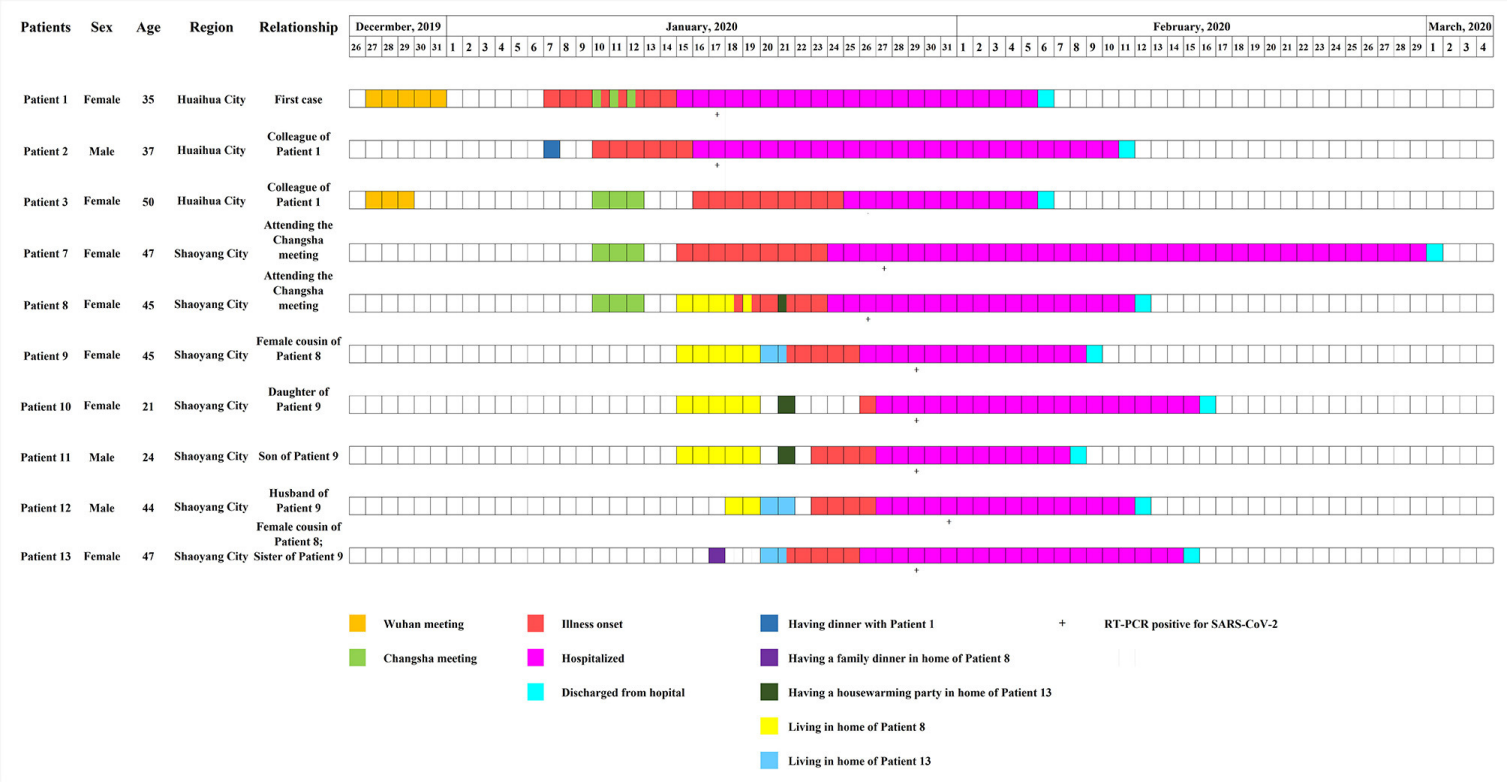
Timeline of severe acute respiratory syndrome coronavirus 2 (SARS-CoV-2) and human adenovirus (HAdV) co-infections. Boxes of different colors specify critical events experienced by patients. From January 15 to 19, 2020, Patient 9, with both her daughter (Patient 10) and son (patient 11), stayed in the home of her cousin (Patient 8). From January 20 to 21, 2020, a couple (Patient 9 and Patient 12) lived in a new house purchased by the sister (Patient 13) of Patient 9. On January 21, a housewarming party attended by Patients 8–12 was held.

On January 15, 2020, the first patient (Patient 1) was enrolled. The patient presented to a hospital in Huaihua City, Hunan Province, China with respiratory symptoms and multiple small patchy shadows in bilateral lungs on chest computed tomography (CT) scan (Supplementary Table 1). This patient tested positive for SARS-CoV-2 by using RT-PCR at a local CDC on January 17, 2020. Epidemiological investigation showed that this patient and her colleague (Patient 3) both had a history of travel to Wuhan, China (Figure 1), and had attended an annual meeting held by a company in Wuhan between December 27 and 31, 2019. During the meeting, they had no history of contact with wild animals, or game food. Nor did they visit markets including the Huanan Seafood Wholesale Market. Her colleague (Patient 3) returned to Huaihua from Wuhan on December 29, and she returned on December 31, 2019. 7 days later (January 7, 2020), she felt uncomfortable, but kept dinner plans with a colleague (Patient 2) that evening. Her colleague (Patient 2) developed respiratory symptoms on January 10, 2020 (Figure 1), but her colleague (Patient 3) did not yet display symptoms.

**Figure 2:**
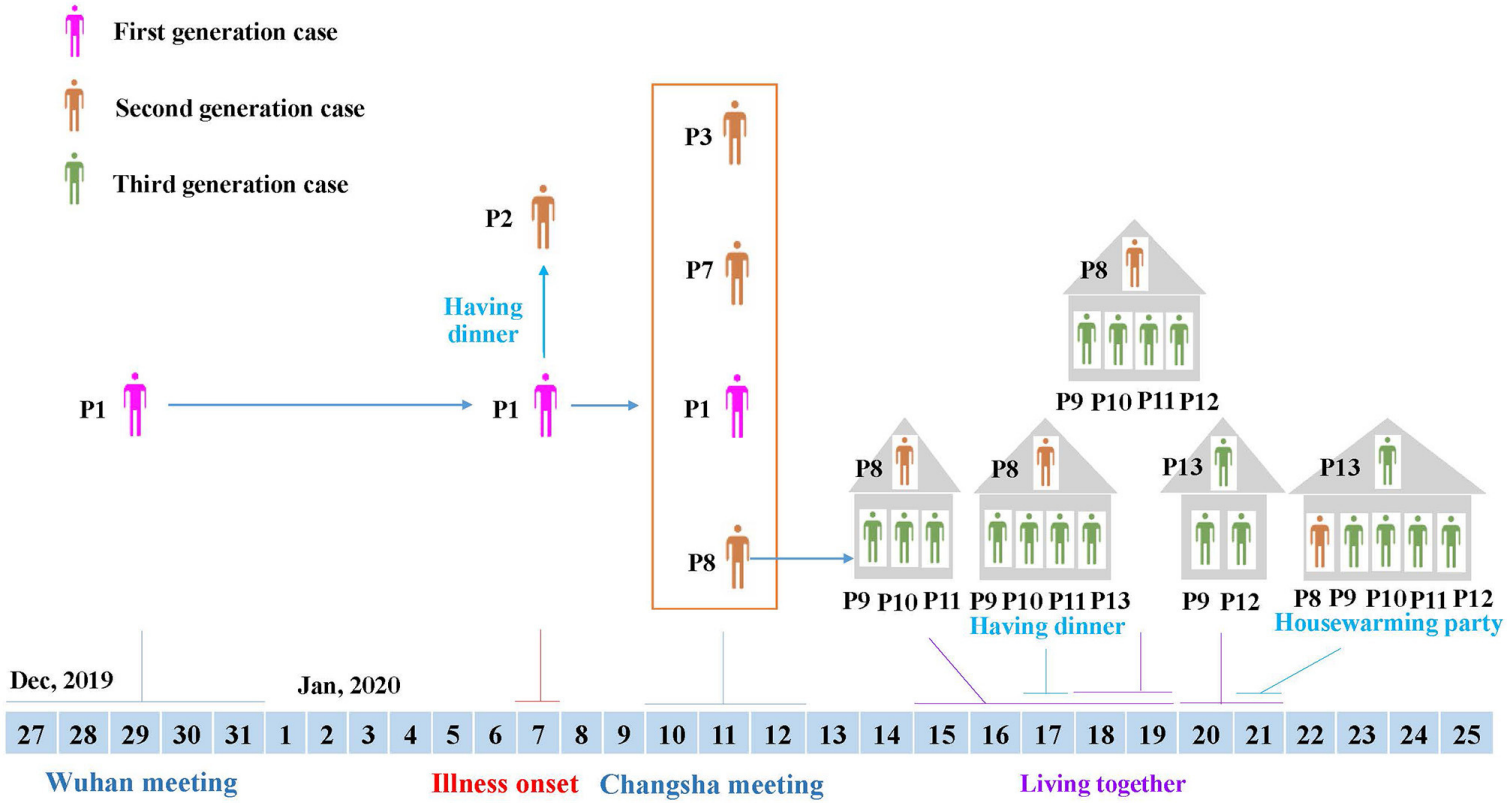
Transmission linkage of the patients. Different colors of individuals indicate the first to third generation cases. Patient 1, the first-generation case identified, was from Huaihua City, and had travel history to Wuhan, where she attended a large conference from December 27 to 31, 2019. After returning home, she developed respiratory symptoms on January 7, 2020, and transmitted the virus to four second-generation cases during a dinner (Patient 2) and meeting (Patient 3, Patient 7 and Patient 8). Then, Patient 8 from Shaoyang City transmitted the virus to her relatives through close contact, resulting in third-generation cases (Patient 5, Patient 9, Patient 10, Patient 11, Patient 12, and Patient 13).

Patient 1, with her colleague (Patient 3), attended another meeting in Changsha City, Hunan Province, China, between January 10 and January 12, 2020 (Figures 1, 2). During the meeting, 282 participants stayed in the same hotel, and gathered in a large conference room. After the meeting, on January 12, 2020, Patients 1 and 3 returned from Changsha to Huaihua and Patient 3 developed respiratory symptoms on January 16, 2020.

Meanwhile, after the Changsha meeting, another two participants (Patients 7 and 8) returned from Changsha to Shaoyang City on January 12, 2020, and showed respiratory illnesses on January 15 (Patient 7), and January 18 (Patient 8), 2020, respectively (Figure 1). Patient 8 was in close contact with relatives, including female cousins (Patients 9 and 13), and her cousins' family members. From January 15 to January 19, 2020, Patient 9 stayed in the house of Patient 8 with her daughter (Patient 10) and son (Patient 11) (Figures 1, 2). On January 17, Patient 13 (female cousin of Patient 8; sister of Patient 9) had a family dinner with Patients 8–11 in the home of Patient 8 (Figure 2). From January 18 to January 19, Patient 12 (husband of Patient 9) also stayed in the house of Patient 8 (Figure 2). In order to celebrate moving into a new house, Patient 13 invited her sister (Patient 9) and her sister's husband (Patient 12) to live in her new house from January 20 to January 21, 2020. On January 21, 2020 she invited her relatives including Patient 8 and her sister's family members (Patients 9, 10, 11, and 12) to a housewarming party (Figures 1, 2). Both Patients 9 and 13 showed respiratory symptoms on January 21, while Patients 11 and 12 showed symptoms on January 23, and Patient 10 became symptomatic on January 26, 2020 (Figure 1).

Among the 10 patients mentioned, three were male and seven were female, and their ages ranged between 21 and 50 years (Supplementary Table 1). The most common symptoms observed were cough (100%), fever (90%), sputum production (70%), shortness of breath (70%), fatigue (40%), and sore throat (30%), followed by diarrhea (20%), chills (10%), rhinorrhea (10%), nasal congestion (10%), nausea (10%), and chest pain (10%). Four patients had chronic comorbidities, including hypertension, coronary disease, and nasosinusitis. All patients showed multiple patchy ground-glass opacities, and most were involved with bilateral lungs on chest CT scans. Blood tests revealed that most patients had normal level of white-cell count, but 60% had lymphopenia. According to the clinical and laboratory results, one was classified as a severe case, five as moderate and four as mild (Supplementary Table 1). All patients were treated with antiviral therapy with Ribavirin (500 mg per dose, 2–3 times daily) and Kaletra (lopinavir-ritonavir, 400/100 mg per dose, 2 times daily) and anti-infection therapy with moxifloxacin. All patients recovered and were discharged on March 1, 2020 (Figure 1). A total of 352 close contacts were traced, and no respiratory symptoms were observed during the medical observation period.

### Laboratory and Genomic Investigation of Patient Samples

**Figure 3:**
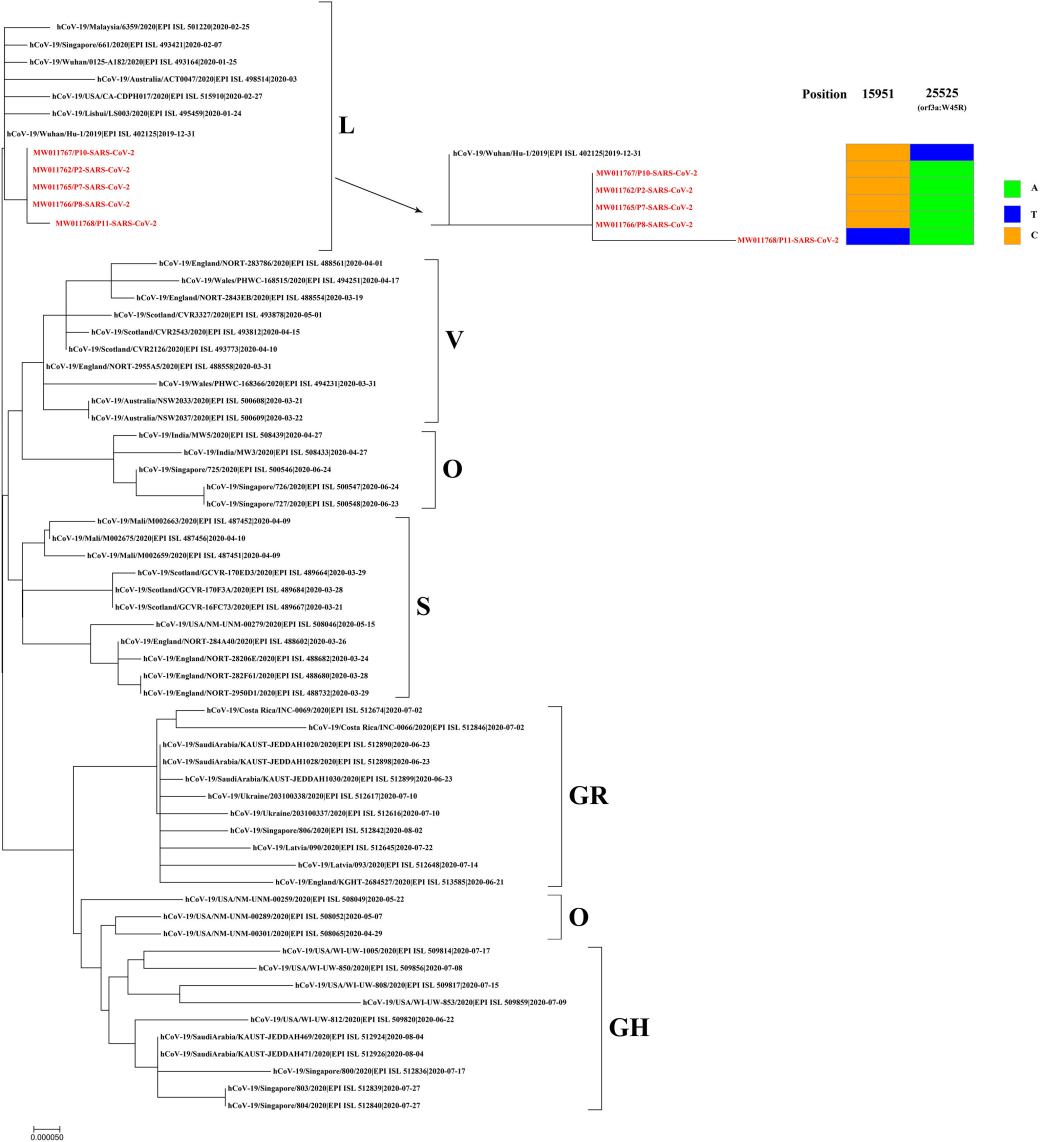
Phylogenetic analysis and sequence comparison of the SARS-CoV-2 genomes sequenced in this study. Phylogenetic analysis was performed based on five complete SARS-CoV-2 genomes sequenced in this study (marked in red) and 57 representative genomes from different SARS-CoV-2 clades. Single nucleotide polymorphisms (SNPs) from the SARS-CoV-2 genomes sequenced in this study are presented in comparison to the first published genome, Wuhan-Hu-1 (GenBank no. NC_045512). New SARS-CoV-2 genomes have been deposited in GenBank, and the accession numbers are given in red.

**Figure 4:**
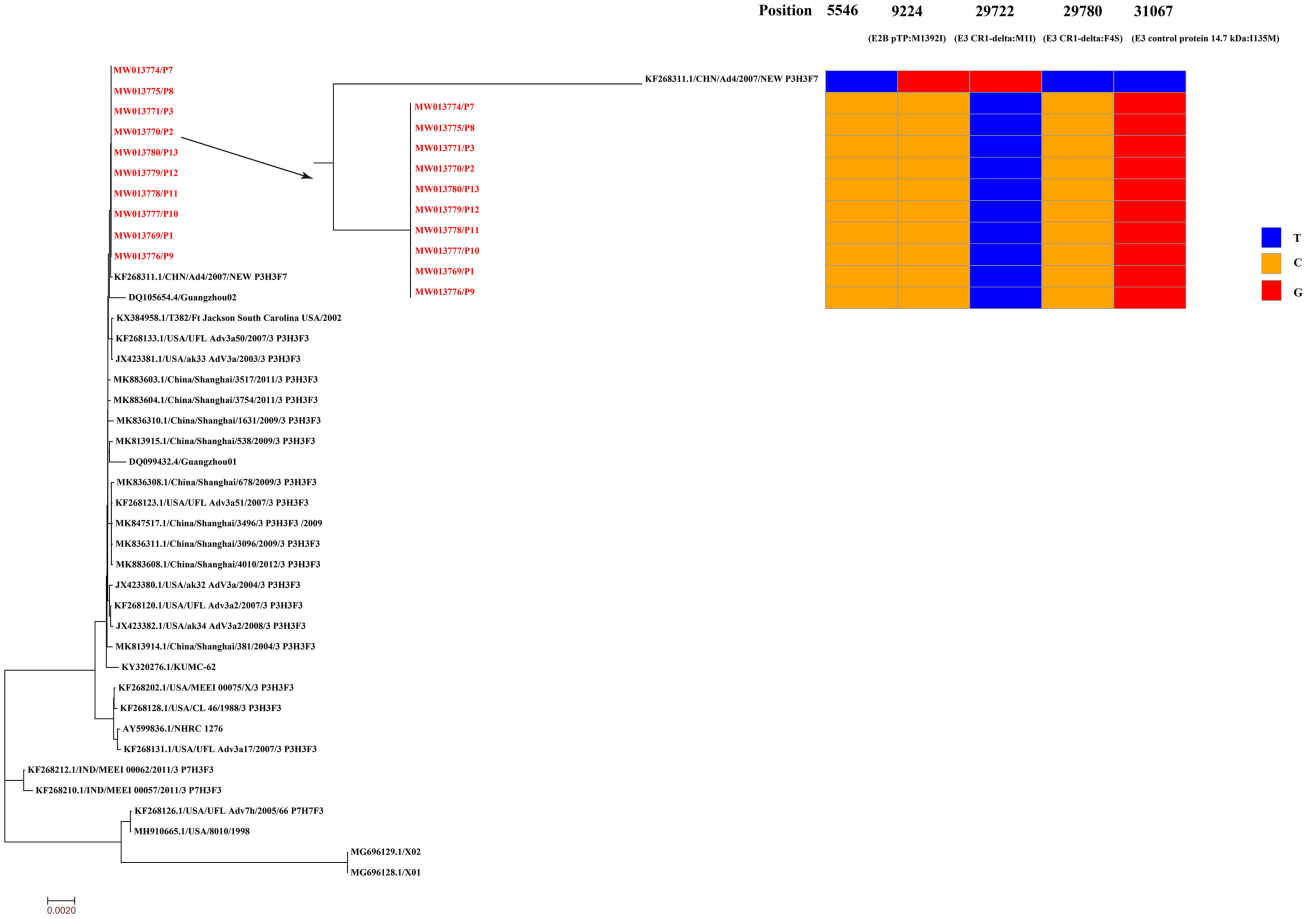
Phylogenetic analysis and sequence comparison of the HAdV genomes sequenced in this study. Phylogenetic analysis was performed based on 10 complete HAdV genomes sequenced in this study (marked in red) and 30 representative HAdV genomes retrieved from GenBank. The HAdV genomes sequenced in this study were determined to be closely related to a reference genome (GenBank no. KF268311) identified in Guangzhou City, China in 2007. Single nucleotide polymorphisms (SNPs) from the HAdV genomes sequenced in this study are presented in comparison to the reference genome KF268311. The HAdV-3 genomes have been deposited in GenBank, and their accession numbers are indicated in red.

Respiratory samples from the 10 patients were detected positive for SARS-CoV-2 via RT-PCR, and samples from 352 of their close contacts were negative for SARS-CoV-2. Sequences of SARS-CoV-2 were obtained directly from respiratory samples using metagenomic sequencing, and unexpectedly HAdV sequences were also identified. RT-PCR assays confirmed that the samples were positive for SARS-CoV-2 and HAdV, but negative for influenza A and B viruses. Using combined whole-genome and Sanger sequencing, we obtained five complete genomes of SARS-CoV-2 and 10 genomes of HAdV. The SARS-CoV-2 and HAdV genomes have been deposited in GenBank under accession nos: MW011762, MW011765-MW011768 for SARS-CoV-2; MW013769-MW013771, MW013774-MW013780 for HAdV. Phylogenetic analysis revealed that the SARS-CoV-2 genomes sequenced in this study clustered together with the early identified SARS-CoV-2 sequences, including the first published SARS-CoV-2 genome, Wuhan-Hu-1 (GenBank no. NC_045512) (Figure 3), which were located in the original lineage (clade L) of SARS-CoV-2 (18). Compared with the first published SARS-CoV-2 genome Wuhan-Hu-1 (GenBank no. NC_045512), two single nucleotide polymorphisms (SNPs) were identified, including one nonsynonymous SNP (W45R) in the orf3a gene and one synonymous SNP in the orf1ab gene. The complete genomes of Patient 2, Patient 7, Patient 8, Patient 10, and Patient 11 all had the W45R mutation in the orf3a gene, and additionally the genome from Patient 11 had a synonymous mutation in the orf1ab gene (Figure 3; Supplementary Table 2). The HAdV genomes sequenced in this study were identified as genotype 3 (HAdV-3), and they clustered together to form a distinct subclade with other HAdV-3 genomes (Figure 4). The HAdV-3 genomes sequenced in this study were determined to be closely related to a genome (GenBank no. KF268311) which was identified in Guangzhou City, China in 2007. Comparative genomic analysis showed that no SNPs were identified among the HAdV-3 genomes sequenced in this study (Figure 4). However, when compared with the reference genome KF268311, five SNPs were identified, including three non-synonymous SNPs (M1I, F4S, I135M) in the E3 gene, one non-synonymous SNP (M1392I) in the E2B gene, and one synonymous SNP in the E2B (Figure 4; Supplementary Table 2).

## Discussion

The most important finding of the present study is that we described an example of co-infection with SARS-CoV-2 and HAdV among some pneumonia patients with obvious epidemiological relationship. Previous studies have reported sporadic cases caused by co-infections of SARS-CoV-2 with other pathogens, including influenza A virus, respiratory syncytial virus, metapneumovirus, rhinovirus, and HAdV (8–12). The detection of SARS-CoV-2 co-infection with other respiratory viruses is difficult. Metagenomic sequencing has shown great potential for the identification of co-infections. When samples positive for SARS-CoV-2 were sent to our laboratory, we performed metagenomic sequencing directly on throat swab samples. Unexpectedly, the sequences of SARS-CoV-2 and HAdV were identified directly from the respiratory samples, and the viral genomes detected within patients clustered together, displaying similar genetic characteristics. These results demonstrated that co-infection with SARS-CoV-2 and HAdV had occurred. Such infections have the potential to result in a cluster of pneumonia patients, and pose unique challenge in the control of the COVID-19 pandemic.

Also of importance are the complex transmission dynamics of co-infection with SARS-CoV-2 and HAdV that are revealed, which resulted in multiple waves of virus transmission through meetings, household interactions, and dinner parties. The first enrolled patient (Patient 1) had traveled from Huaihua City to Wuhan City with her colleague (Patient 3) to attend a conference. She returned to Huaihua City later, but developed respiratory symptoms earlier than her colleage, within the estimated incubation period of SARS-CoV-2 (19). The HAdV-3 genome from Patient 1 was identical to those from other patients, and similar to the genome identified in 2007 in China. HAdV-3 is one of the most prevalent serotypes of HAdV globally, especially in Asia, and is commonly associated with epidemic infections among civilian and military populations (20). Moreover, the SARS-CoV-2 genome from Patient 1 was closely related to the first published genome Wuhan-Hu-1, and it was located in the original L clade (18), indicative of its likely Wuhan origin. Therefore, we believe that Patient 1 was the first to be infected among the patients considered, and probably acquired the infection while attending a meeting in Wuhan.

During her illness, Patient 1 had contact with her colleague (Patient 2) at a dinner, and attended the Changsha meeting with another colleague (Patient 3). Although Patients 1 and 3 all attended the Wuhan meeting and Changsha meeting, Patient 3 returned home from Wuhan earlier than Patient 1, and the timing of respiratory symptom onset was beyond the maximum incubation period of SARS-CoV-2 (6, 19). This suggested that Patient 3 acquired the infection during the Changsha, and not Wuhan meeting. Notably, two persons (Patients 7 and 8) from Shaoyang City also attended the Changsha meeting and lived in the same hotel as Patient 1. After returning home, they developed respiratory illness within the incubation period of SARS-CoV-2. These results suggested that Patient 1 was the first case, who acquired an infection during the Wuhan meeting or travel, and subsequently transmitted the virus to at least four second-generation cases (Patients 2, 3, 7, and 8) (Figure 2). Thereafter, Patient 8 who lived in Shaoyang City, transmitted the virus to family membersvia a family gathering, which resulted in third-generation cases (Figure 2). Since SARS-CoV-2 has emerged in Wuhan, thousands of exported and resultant secondary cases have been reported in multiple countries, and many infection clusters have been identified, mostly among secondary cases (5, 7, 21–24). Our present study provides a clear example of the persistent transmission of co-infection with SARS-CoV-2 and HAdV, which can result in at least third-generation cases.

Symptom-based screening of suspected SARS-CoV-2 patients may fail to identify all SARS-CoV-2 infections. Currently, the asymptomatic and presymptomatic transmission of SARS-CoV-2 infections have been reported, which are challenges to effective disease control (21–27). In this study, we also observed the presymptomatic transmission of SARS-CoV-2 and HAdV co-infection. Patient 8 attended the Changsha meeting, and she developed respiratory symptoms 6 days later. During the virus incubation period, she was in close contact with four of her relatives (Patients 9–12) through family clustering. The family members developed illnesses 3, 5, and 8 days later. Patient 13 attended a family dinner with Patient 8 and her sister's family members (Patients 9–11) when they were all presymptomatic. 4 days later, Patient 13 was ill. Thus, attendees likely acquired infections from Patient 8, a presymptomatic case who was shedding the virus but not yet showing symptoms. The present study, in combination with previous studies, demonstrates that presymptomatic COVID-19 cases are infectious, and have resulted in presymptomatic transmission and clusters of patients (22, 24–26). Notably, a recent study by Zhu *et al*. showed that SARS-CoV-2 has a high rate of co-infection rate with other pathogens, which was observed in patients 4–0 days after symptom onset. This indicates that presymptomatic individuals have a great potential to transmit co-infection (8). Further, presymptomatic individuals are potentially contagious, highlighting the role of presymptomatic cases in mediating viral expansion. Especially in family clusters, family members should be closely monitored and carefully examined to rule out infection, even if they test negative for SARS-CoV-2 or are asymptomatic, to prevent and control the further transmission of the virus.

In this study, the patients identified showed clinical symptoms similar to those that have been described in previous reports (1, 6, 28). They had typical characteristics of SARS-CoV-2 infections, including lymphopenia and bilateral ground-glass opacity presence (1, 6). Most patients were defined as moderate or mild cases, and only one severe case was identified. Further, all patients recovered. HAdV species B is reported to be one of the primary causes of acute respiratory disease, and can cause severe illnesses, particularly in children and individuals with underlying health conditions. HAdV is also commonly associated with acute respiratory disease outbreaks, especially in military and hospital settings (20, 29, 30). The study by Zhu *et al*. reported that patients co-infected with SARS-CoV-2 and HAdV tended to be 15–65 years of age, with mild symptoms (8). Thus, co-infection seemingly did not aggravate disease (11). However, further studies and comprehensive surveillance are needed to determine the role of co-infection in the mediation of illness severity in patients with SARS-CoV-2.

This study has several limitations. Only 10 patients with co-infection of SARS-CoV-2 and HAdV were identified and included. All patients presented to the hospitals after symptom onset. The patients were distributed over two cities, and involved two Provinces of China. Detailed epidemiological data are difficult to obtain, and systematic sample collection early during the course of illness was limited. Thus, the limitations of this study are its flaws or shortcomings which could be the result of unavailability of resources, small sample size, flawed methodology, etc.

In summary, we identified some pneumonia patients with obvious epidemiological relationship, which were caused by co-infection with SARS-CoV-2 and HAdV. This study provides a clear example of the persistent transmission of SARS-CoV-2 and HAdV co-infection, which spread inter-regionally and throughout third generations of patients. Our findings also indicate that co-infection with SARS-CoV-2 and HAdV can be latently spread, a characteristic that makes controlling the COVID-19 pandemic especially challenging. Therefore, continuous surveillance is needed to monitor the prevalence, infectivity, transmissibility, and pathogenicity of pathogen-SARS-CoV-2 co-infection to evaluate the precise risk associated with co-infections.

## Data Availability Statement

The datasets presented in this study can be found in online repositories. The names of the repository/repositories and accession number(s) can be found at: GenBank under accession numbers: MW011762, MW011765-MW011768 for SARS-CoV-2; MW013769-MW013771, MW013774-MW013780 for HAdV.

## Author Contributions

SQ, SH, HS, and ZZ contributed to the conception and design of the study. GZ, XL, HZ, XX, XC, GY, LG, SH, and ZZ carried out sample collection and data acquisition. SQ, GZ, HL (Seventh Author), XD, HL (Fifth Author), HZ, XX, HW, ST, MY, and LG performed the laboratory sample analysis. SQ, PL, and LW performed statistical analyses. SQ, PL, and ZZ wrote the first draft of the manuscript. HS and ZZ supervised this study. All authors approved the final manuscript as submitted, and agreed to be accountable for all aspects of the work.

## Funding

This work was supported in part by the National Science and Technology Major Project of China (2018ZX10101003 and 2018ZX10714002), Hunan Provincial Construction of Innovative Provinces Special Social Development Areas Key Research and Development Project (2020SK3012), and the Chinese Academy of Medical Sciences Coronavirus Disease 2019 Science and Technology Research Project in 2020 (2020HY320003).

## Conflict of Interest

The authors declare that the research was conducted in the absence of any commercial or financial relationships that could be construed as a potential conflict of interest.

## Publisher's Note

All claims expressed in this article are solely those of the authors and do not necessarily represent those of their affiliated organizations, or those of the publisher, the editors and the reviewers. Any product that may be evaluated in this article, or claim that may be made by its manufacturer, is not guaranteed or endorsed by the publisher.
